# Elevated Osteopontin and Interferon Gamma Serum Levels and Increased Neutrophil-to-Lymphocyte Ratio Are Associated With the Severity of Symptoms in Schizophrenia

**DOI:** 10.3389/fpsyt.2019.00996

**Published:** 2020-01-23

**Authors:** Márton Áron Kovács, Tamás Tényi, Réka Kugyelka, Lilla Prenek, Lídia Hau, Éva Erzsébet Magyar, Róbert Herold, Péter Balogh, Diána Simon

**Affiliations:** ^1^ Department of Psychiatry and Psychotherapy, Clinical Center, University of Pécs Medical School, Pécs, Hungary; ^2^ Department of Immunology and Biotechnology, Clinical Center, University of Pécs Medical School, Pécs, Hungary

**Keywords:** osteopontin, cytokines, schizophrenia, the Positive and Negative Syndrome Scale, inflammation, antipsychotics

## Abstract

Inflammation and immune dysregulation could contribute to the pathogenesis of schizophrenia. Osteopontin (OPN) is a cytokine-like glycoprotein involved in inflammation and in modulating immune responses, and it can also directly modify the cytokine expression and survival of microglia. Furthermore, elevated gene expression of OPN in first episode psychosis has recently been described, but to date OPN level has not been investigated in schizophrenia. Imbalance of T-helper subtypes could also represent a vulnerability factor for schizophrenia. In this study, we analyzed the concentration of OPN, levels of cytokines associated with T-helper subtypes: interferon gamma (IFNy) for Th1, interleukin (IL)-10 for Th2, IL-8 for Th17, and neutrophil-to-lymphocyte ratio (NLR) in 22 patients with schizophrenia assessed for the intensity of their symptoms by the Positive and Negative Syndrome Scale (PANSS) and Clinical Global Impression scale (CGI) scores. Serum OPN, IFNy, IL-10, and IL-8 concentrations were measured by ELISA kits and NLR was calculated from blood count. We found significant correlation between the level of OPN and PANSS-total and PANSS-general scores. IFNy level and NLR showed significant correlation with PANSS-total, PANSS-positive, PANSS-general, and CGI score. Among the measured markers antipsychotic therapy only had significant effects on NLR and OPN level, both of which were significantly reduced after long-term antipsychotic treatment. Our results indicate that elevated OPN and IFNy concentrations, and increased NLR are associated with severe symptoms in schizophrenia and suggest the importance of Th1 subtype in patients with high PANSS-positive and PANSS-general subscore. Significant correlation between NLR and PANSS scores strengthens the inflammation hypothesis of schizophrenia.

## Introduction

Neurobiological and neuroimaging studies suggested that immunological and inflammatory aspects should be considered as potential contributors to the pathophysiology of schizophrenia (1). The imbalance of cytokines can cause neuronal damages and could contribute to the pathogenesis of neuropsychiatric disorders (2–6). Osteopontin (OPN) is considered a key cytokine in cellular immune response and may also regulate inflammation (7, 8). OPN concentration is elevated in central nervous system lesions in ischemic and traumatic brain injuries, indicating that it may contribute to the neuropathology in neurodegenerative diseases (9, 10). One possible mechanism may be related to OPN’s capacity to directly modulate the cytokine expression and survival of microglia (11). Furthermore, elevated gene expression of OPN in first episode psychosis (FEP) has recently been described (12). Neutrophil-to-lymphocyte ratio (NLR) is a simple and inexpensive marker of systemic inflammation. The inflammation hypothesis of schizophrenia is supported by the significant increase of NLR both in FEP patients and in chronic cases (13). Immune dysregulation could represent a vulnerability factor for schizophrenia (14, 15) and Th1 and Th2 imbalance is a reported immunological alteration in schizophrenia patients (16, 17). The activation of Th17 cells has also been described in first episode schizophrenia (18) and the Th17 pathway has been suggested to play a role in the pathogenesis of the disease (19).

The aim of this study was to find possible associations between serum OPN level and severity of symptoms, since to date, the concentration of OPN has not been measured in schizophrenia patients. Our aim also was to measure the concentrations of signature cytokines interferon gamma (IFNy) for Th1 cells, interleukin 10 (IL-10) for Th2 subset, and interleukin 8 (IL-8) for Th17 lymphocytes, respectively, in serum samples and to calculate NLR from blood counts to evaluate the relevance of T-helper subtypes and NLR in the assessment of the severity of schizophrenia.

## Methods

### Patients

Altogether 22 patients treated at the Department of Psychiatry and Psychotherapy at the University of Pécs, Hungary were included in the study (**Table 1**). All patients were diagnosed with schizophrenia according to the Diagnostic and Statistical Manual of Mental Disorders, Fifth Edition (DSM-5) and underwent a comprehensive psychiatric evaluation and an assessment of acute psychotic exacerbation by the Positive and Negative Syndrome Scale (PANSS) and Clinical Global Impression scale (CGI). All patients were on antipsychotic medication during the study. Acute or chronic somatic comorbidities (allergies, autoimmune disorders, cancer, fever, infection) were exclusion criteria. NLR was calculated from the blood count. All patients agreed to participate in the study and signed a written informed consent form. The study was approved by the Regional Clinical Research Ethics Committee (5951—PTE 2015).

**Table 1:** Patients’ characteristics.

	Schizophrenia patients (n = 22)
Age	49 ± 10.21
Sex (male)	13 (59.09%)
Family history (positive)	8 (36.4%)
Smoking habits (yes)	13 (59.09%)
Marital status (not married)	21 (95.5%)
Disease duration (years)	23.6 ± 7.49
Length of hospitalization (weeks)	3.29 ± 1.27
BMI (kg/m^2^)	26.6 ± 2.3
Cholesterol (mmol/l)	4.8 ± 1.1
Triglyceride (mmol/l)	1.5 ± 0.83
**Anti-psychotic therapy**	
Number of drugs (one/more)	5 (22.73%)/17 (77.27%)
Type of therapy (first generation/second generation/combined)	1 (4.55%)/11 (50%)/10 (45.45%)
Length of therapy (short-term/long-term)	3.4 ± 1.81 weeks (n = 11)/8.82 ± 5.95 years (n = 11)
**Clinical parameter**	
CGI	4.045 ± 0.95
PANSS-total	71.91 ± 15.61
PANSS-general	33.95 ± 10.11
PANSS-negative	19.73 ± 3.03
PANSS-positive	18.23 ± 5.81

CGI, Clinical Global Impression; PANSS, Positive and Negative Syndrome Scale. Data are presented as n% or mean ± SD.

### Measurement of Osteopontin, IFNy, IL-10, and IL-8 Levels in Peripheral Blood of Schizophrenia Patients

Peripheral blood was drawn and the serum concentration of the markers was quantified using Human ELISA sets (OPN and IL10: Bio-Techne, Minneapolis, MN, USA; IFNy and IL-8: BD Biosciences, Franklin Lakes, NJ, USA) according to the manufacturer’s protocol. The reaction was developed with TMB and measured at 450 nm using an iEMS MF microphotometer (Thermo Labsystem, Beverly MA, USA).

### Statistical Analysis

Statistical evaluation was performed with SPSS v. 25.0 statistics package (IBM, USA). To test the distribution of variables Shapiro-Wilk normality test was used due to the small number of cases. Continuous variables were compared with the Mann-Whitney U test or Student’s t test. Relationship between continuous variables was assessed with Spearman correlation. A p value < 0.05 was considered significant.

## Results

**Figure 1:**
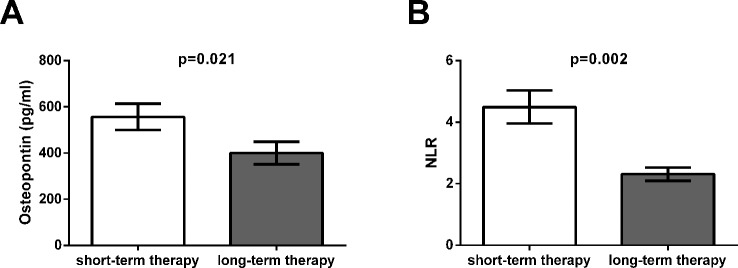
Significant effects of antipsychotic treatment on the serum concentration of osteopontin (OPN) and neutrophil-to-lymphocyte ratio (NLR). **(A)** The serum concentration of OPN was significantly decreased (p = 0.021) in patients on long-term antipsychotic therapy (8.8 ± 5.9 years) compared to patients on short-term therapy (3.5 ± 1.9 weeks). **(B)** NLR was also significantly decreased (p = 0.002) in patients on long-term antipsychotic therapy (8.8 ± 5.9 years) compared to patients on short-term therapy (3.5 ± 1.9 weeks).

Significant positive correlation was found between the concentration of OPN and the severity of symptoms measured by PANSS-total and PANSS-general scores. Furthermore, IFNy level and NLR showed significant positive correlation with PANSS-total, PANSS-positive, PANSS-general and CGI score (**Table 2**). Serum concentration of OPN also showed significant correlation with NLR (p = 0.005 and r = 0.598). Among the measured markers the applied antipsychotic therapy only had significant effects on the concentration of OPN and NLR. Patients on long-term antipsychotics treatment had significantly lower NLR (p = 0.002) and OPN level (p = 0.021) compared to patients on short-term therapy (**Figure 1**). It is interesting to note, that the only patient on risperidone monotherapy had outlier values of the cytokines thus had to be excluded from the statistical analysis. Risperidone, when used in combination with other antipsychotics had no significant effect on the concentration of any cytokines tested. There were no significant association of concentrations of IL-10, IL-8 with clinical data. Age, gender, disease duration, smoking, BMI, cholesterol, and triglyceride levels did not influence the levels of the investigated markers.

**Table 2:** Correlations among clinical parameters and serum interferon gamma (IFNγ), osteopontin (OPN) concentrations, and neutrophil-to-lymphocyte ratio (NLR).

Clinical parameters		IFNγ	OPN	NLR	NLR
CGI	Correlation coefficient	0.524	0.340	0.506	0.506
	***p value***	0.018	0.142	0.019	0.019
PANSS-total	Correlation coefficient	0.536	0.563	0.594	0.594
	***p value***	0.015	0.010	0.005	0.005
PANSS-general	Correlation coefficient	0.616	0.526	0.543	0.543
	***p value***	0.004	0.017	0.011	0.011
PANSS-negative	Correlation coefficient	−0.211	0.158	0.227	0.227
	***p value***	0.371	0.505	0.322	0.322
PANSS-positive	Correlation coefficient	0.496	0.417	0.552	0.552
	***p value***	0.026	0.067	0.009	0.009

CGI, Clinical Global Impression; PANSS, Positive and Negative Syndrome Scale; IFNγ, interferon gamma; OPN, osteopontin; NLR, neutrophil-to-lymphocyte ratio.

## Discussion

In this study we have measured the concentration of OPN in peripheral blood of schizophrenia patients that has, to our knowledge, so far eluded scrutiny in this disease, and we found that serum OPN level correlated significantly with the severity of symptoms measured by PANSS-total and PANSS-general scores. OPN has been described to enhance Th1 response (7), thus might have an additional effect in the Th1 deflection in schizophrenia patients with elevated concentration of IFNy. According to our results, serum concentration of OPN showed significant correlation with NLR, suggesting that its immunomodulatory effect may support the inflammatory response in schizophrenia. We were the first to analyze the possible effects of antipsychotic treatment on the serum level of OPN and found that years of treatment with antipsychotics significantly reduced the serum level of OPN. It is noteworthy that in our study the only patient, who was on risperidone monotherapy had an extremely high concentration of OPN. This is in agreement with the results of the recent study, which found that on risperidone monotherapy, the messenger RNA (mRNA) expression of OPN was significantly upregulated (12). We also found that NLR correlated significantly with PANSS-total score, which was described by Kulaksizoglu, B. and Kulaksizoglu, S. (20). Additionally, according to our results NLR also showed significant correlation with PANSS-positive and PANSS-general and CGI scores. These findings suggest an association between NLR and the severity of symptoms in schizophrenia., furthermore our results demonstrated that years of antipsychotic treatment significantly reduced NLR. Cytokines can be considered potential state or trait markers in schizophrenia patients (21, 22). Serum concentration of IFNy is elevated in schizophrenia (23), but results are contradictory whether IFNy can be considered a trait marker. We found that IFNy level showed a significant correlation with PANSS-total, PANSS-positive, PANSS-general subscores, and CGI score, thus IFNy level could be an indicative marker of disease severity in schizophrenia. IL-8 is an inflammatory chemokine significantly upregulated in the dorsolateral prefrontal cortex of individuals with schizophrenia (24) and a peripheral inflammatory biomarker found in FEP patients (25) and in multiple-episode schizophrenia (MES) patients (24). A significant positive correlation was shown between serum concentration of IL-8 and PANSS negative subscale in neuroleptic-free schizophrenia patients (26). We were unable to detect any significant correlation between the level of IL-8 and PANSS scales in antipsychotic treated patients. Elevated level of IL-10 was measured in FEP (27) and MES patients (23). On the contrary, IL-10 was also reported to be decreased in FEP and showed inverse correlation with PANSS-negative subscale (28). However, a recent study showed no correlation between IL-10 concentration and PANSS score (29), which is in agreement with our results. According to Noto et al. (27) after risperidone treatment, IL-10 concentration decreased significantly and it is interesting to note that we were unable to detect IL-10 in the serum sample of the patient on risperidone monotherapy. Nevertheless, we found that risperidone, when used in combination with other antipsychotics, had no significant influence on the level of IL-10 or OPN. Schizophrenic patients on antipsychotic treatment have risk for developing metabolic syndrome and although the underlying concrete mechanisms are still unclear, the length of antipsychotic use may be a risk factor (30), but our results show that BMI, cholesterol, and triglyceride levels were not significantly different in patients on short- and long-term antipsychotic therapy. Furthermore, we also found that OPN, IFNγ, IL-10, IL-8 concentrations, and NLR did not correlate with BMI, cholesterol and triglyceride levels, which supports the theory that apart from antipsychotic therapy and immune factors the genetics and lifestyle of the patients could also have a role in obesity and altered levels of blood fats (30).

Our study has the limitation that healthy controls were not enrolled, but our aim was to correlate the investigated immunological markers with the severity of symptoms in schizophrenia. Overall, we found that the serum concentrations of OPN and IFNy correlated significantly with PANSS-total and PANSS-general scores. Additionally, peripheral blood level of IFNy showed significant correlation with PANSS-positive score suggesting the relevance of Th1 subtype in schizophrenia patients with high PANSS-positive scores also. NLR correlated significantly with PANSS scores strengthening the inflammation hypothesis of schizophrenia. Antipsychotic treatment had significant effects on the level of OPN and on NLR, but not on the level of IFNy. Besides increased NLR, elevated concentrations of OPN and IFNy could reflect the severity of schizophrenia and support the theory of immunopathogenesis in schizophrenia.

## Data Availability Statement

The datasets generated for this study are available on request to the corresponding author.

## Ethics Statement

The studies involving human participants were reviewed and approved by Regional Clinical Research Ethics Committee, University of Pécs. The patients/participants provided their written informed consent to participate in this study.

## Author Contributions 

TT and DS designed the study. MK, RK, LP, and DS performed the experiments. LH, ÉM, RH, and TT contributed the clinical data. MK and DS analyzed the data. MK, PB, and DS wrote the first draft of the manuscript. All authors contributed to and approved the final manuscript.

## Funding

The study was supported by the Hungarian National Brain Research Programme KTIA-13-NAP-A-II/12(2018-2022), the Hungarian National Excellence Centrum Grants (2018–2019, 2019–2020) and the ÚNKP-17-2-I New National Excellence Program of the Ministry of Human Capacities. The work was also supported by the European Union, co-financed by the European Social Fund as part of the project “PEPSYS—Complexity of peptide-signalization and its role in systemic diseases” of GINOP 2.3.2-15-2016-00050 and EFOP 3.6.1-16-2016-00004 grants.

## Conflict of Interest

The authors declare that the research was conducted in the absence of any commercial or financial relationships that could be construed as a potential conflict of interest.

## References

[1] Müller N Inflammation in schizophrenia: pathogenetic aspects and therapeutic considerations. Schizophr Bull (2018) 44(5):973–82. 10.1093/schbul/sby024 PMC6101562 29648618

[2] Goldsmith DR Rapaport MH Miller BJ. A meta-analysis of blood cytokine network alterations in psychiatric patients: comparisons between schizophrenia, bipolardisorder and depression. Mol Psychiatry (2016) 21(12):1696–709. 10.1038/mp.2016.3 PMC6056174 26903267

[3] Kronfol Z Remick DG Cytokines and the brain: implications for clinical psychiatry. Am J Psychiatry (2000) 157(5):683–94. 10.1176/appi.ajp.157.5.683 10784457

[4] Qin L Wu X Block ML Liu Y Breese GR Hong JS Systemic LPS causes chronic neuroinflammation and progressive neurodegeneration. Glia (2007) 55(5):453–62. 10.1002/glia.20467 PMC2871685 17203472

[5] Raison CL Miller AH Is depression an inflammatory disorder? Curr Psychiatry Rep (2011) 13(6):467–75. 10.1007/s11920-011-0232-0 PMC3285451 21927805

[6] Smyth AM Lawrie SM The neuroimmunology of schizophrenia. Clin Psychopharmacol Neurosci (2013) 11(3):107–17. 10.9758/cpn.2013.11.3.107 PMC3897758 24465246

[7] Ashkar S Weber GF Panoutsakopoulou V Sanchirico ME Jansson M Zawaideh S Eta-1 (osteopontin): an early component of type-1 (cell-mediated) immunity. Science (2000) 287(5454):860–4. 10.1126/science.287.5454.860 10657301

[8] Lund SA Giachelli CM Scatena M The role of osteopontin in inflammatory processes. J Cell Commun Signal (2009) 3(3–4):311–22. 10.1007/s12079-009-0068-0 PMC2778587 19798593

[9] Chan JL Reeves TM Phillips LL Osteopontin expression in acute immune response- mediates hippocampal synaptogenesis and adaptive outcome following cortical brain injury. Exp Neurol (2014) 261:757–71. 10.1016/j.expneurol.2014.08.015 PMC4262258 25151457

[10] Yu H Liu X Zhong Y The effect of Osteopontin on Microglia. Biomed Res Int (2017) 2017:1879437. 10.1155/2017/1879437 28698867 PMC5494082

[11] Rabenstein M Vay SU Flitsch LJ Fink GR Schroeter M Rueger MA Osteopontin directly modulates cytokine expression of primary microglia and increases their survival. J Neuroimmunol (2016) 299:130–8. 10.1016/j.jneuroim.2016.09.009 27725111

[12] Mantere O Trontti K García-González J Balcells I Saarnio S Mäntylä T Immunomodulatory effects of antipsychotic treatment on gene expression in first-episode psychosis. J Psychiatr Res (2019) 109:18–26. 10.1016/j.jpsychires.2018.11.008 30463035

[13] Karageorgiou V Milas GP Michopoulos I Neutrophil-to-lymphocyte ratio in schizophrenia: A systematic review and meta-analysis. Schizophr Res (2019) 206:4–12. 10.1016/j.schres.2018.12.017 30573407

[14] Miller BJ Goldsmith DR Towards an immunophenotype of schizophrenia: progress, potential mechanisms, and future directions. Neuropsychopharmacology (2017) 42(1):299–317. 10.1038/npp.2016.211 27654215 PMC5143505

[15] Pape K Tamouza R Leboyer M Zipp F Immunoneuropsychiatry - novel perspectives on brain disorders. Nat Rev Neurol (2019) 15(6):317–28. 10.1038/s41582-019-0174-4 30988501

[16] Müller N Riedel M Ackenheil M Schwarz MJ The role of immune function in schizophrenia: an overview. Eur Arch Psychiatry Clin Neurosci (1999) 249 Suppl 4:62–8. 10.1007/pl00014187 10654111

[17] Müller N Riedel M Ackenheil M Schwarz MJ Cellular and humoral immune system in schizophrenia: a conceptual re-evaluation. World J Biol Psychiatry (2000) 1(4):173–9. 10.3109/15622970009150588 12607212

[18] Ding M Song X Zhao J Gao J Li X Yang G Activation of Th17 cells in drug naïve, first episode schizophrenia. Prog Neuropsychopharmacol Biol Psychiatry (2014) 51:78–82. 10.1016/j.pnpbh.2014.01.001 24447943

[19] Debnath M Berk M Th17 pathway-mediated immunopathogenesis of schizophrenia: mechanisms and implications. Schizophr Bull (2014) 40(6):1412–21. 10.1093/schbul/sbu049 PMC4193719 24711545

[20] Kulaksizoglu B Kulaksizoglu S Relationship between neutrophil/lymphocyte ratio with oxidative stress and psychopathology in patients with schizophrenia. Neuropsychiatr Dis Treat (2016) 12:1999–2005. 10.2147/NDT.S110484 27574431 PMC4991539

[21] Miller BJ Buckley P Seabolt W Mellor A Kirkpatrick B Meta-analysis of cytokine alterations in schizophrenia: clinical status and antipsychotic effects. Biol Psychiatry (2011) 70(7):663–71. 10.1016/j.biopsych.2011.04.013 PMC4071300 21641581

[22] Tomasik J Rahmoune H Guest PC Bahn S Neuroimmune biomarkers in schizophrenia. Schizophr Res (2016) 176(1):3–13. 10.1016/j.schres.2014.07.025 25124519

[23] Frydecka D Krzystek-Korpacka M Lubeiro A Stramecki F Stańczykiewicz B Beszłej JA Profiling inflammatory signatures of schizophrenia: a cross-sectional and meta-analysis study. Brain Behav Immun (2018) 71:28–36. 10.1016/j.bbi.2018.05.002 29730395

[24] Fillman SG Cloonan N Catts VS Miller LC Wong J McCrossin T Increased inflammatory markers identified in the dorsolateral prefrontal cortex of individuals with schizophrenia. Mol Psychiatry (2013) 18(2):206–14. 10.1038/mp.2012.110 22869038

[25] Trovão N Prata J VonDoellinger O Santos S Barbosa M Coelho R Peripheral biomarkers for first-episode psychosis-opportunities from the neuroinflammatory hypothesis of schizophrenia. Psychiatry Investig (2019) 16(3):177–84. 10.30773/pi.2018.12.19.1 PMC6444098 30836740

[26] Zhang XY Zhou DF Zhang PY Wu GY Cao LY Shen YC Elevated interleukin-2, interleukin-6 and interleukin-8 serum levels in neuroleptic-free schizophrenia: association with psychopathology. Schizophr Res (2002) 57(2-3):247–58. 10.1016/S0920-9964(01)00296-1 12223256

[27] Noto C Ota VK Gouvea ES Rizzo LB Spindola LM Honda PH Effects of risperidone on cytokine profile in drug-naïve first-episode psychosis. Int J Neuropsychopharmacol (2014) 18(4):pyu042. 10.1093/ijnp/pyu042 25522386 PMC4360233

[28] Xiu MH Yang GG Tan YL Chen DC Tan SP Wang ZR Decreased interleukin-10 serum levels in first episode drug-naive schizophrenia: relationship to psychopathology. Schizophr Res (2014) 156(1):9–14. 10.1016/j.schres.2014.03.024 24766914

[29] Dahan S Bragazzi NL Yogev A Bar-Gad M Barak V Amital H The relationship between serum cytokine levels and degree of psychosis in patients with schizophrenia. Psychiatry Res (2018) 268:467–72. 10.1016/j.psychres.2018.07.041 30138859

[30] Jeon SW Kim YK Unresolved issues for utilization of atypical antipsychotics in schizophrenia: antipsychotic polypharmacy and metabolic syndrome. Int J Mol Sci (2017) 18(10):E2174. 10.3390/ijms18102174 29057817 PMC5666855

